# Cholinergic Signaling Alters Stress-Induced Sensitization of Hippocampal Contextual Learning

**DOI:** 10.3389/fnins.2019.00251

**Published:** 2019-03-19

**Authors:** Sarah Hersman, Ann N. Hoffman, Liliann Hodgins, Shannon Shieh, Jamie Lam, Ashen Parikh, Michael S. Fanselow

**Affiliations:** Departments of Psychology, Neurobiology, Psychiatry and Biobehavioral Sciences, and Integrative Center for Learning and Memory, University of California, Los Angeles, Los Angeles, CA, United States

**Keywords:** stress-enhanced fear learning, sensitization, acetylcholine, scopolamine, fear learning, amygdala, hippocampus

## Abstract

Post-traumatic stress disorder (PTSD) has a profound contextual component, and has been demonstrated to alter future contextual learning. However, the mechanism by which a single traumatic event affects subsequent contextual experiences has not been isolated. Acetylcholine (ACh) is an important modulator of hippocampus-dependent learning such as contextual memory strength. Using Stress-Enhanced Fear Learning (SEFL), which models aspects of PTSD in rats, we tested whether muscarinic acetylcholine receptors (mAChR) in dorsal hippocampus (DH) are required during trauma for the effect of trauma on subsequent contextual fear learning. We infused scopolamine or vehicle into DH immediately before stress, and tested fear in both the trauma context and a novel context after a mild stressor. The results show that during learning, ACh acting on mAChR within the DH is required for sensitization of future contextual fear learning. However, this effect is selective for contextual learning, as this blockade leaves discrete cue sensitization intact. Rather than simply sensitizing the BLA, as previous studies have suggested, SEFL requires cholinergic signaling in DH for contextual sensitization.

## Introduction

For fear to be adaptive, it must be titrated to the level of threat and relatively specific for threat-related stimuli. Both aspects of this fear responding are dysregulated in anxiety disorders such as post-traumatic stress disorder (PTSD), disorders where fear responses are enhanced and disrupt an individual’s normal functioning (Rosen and Schulkin, 1998; Bonne et al., 2004). Though the neural mechanisms for human PTSD generation are currently unknown and likely to be heterogeneous (Roozendaal et al., 2009; Bennett et al., 2015), circuit models of PTSD implicate the amygdala as an important structure for storage of traumatic memories and the influence of stress on emotional memory acquisition (Rosen and Schulkin, 1998; Waddell et al., 2008). In these models, acute or chronic stress leads to a “hyperactive” amygdala, manifested by increased excitability of glutamatergic principal cells or reduced inhibitory drive from GABAergic inhibitory interneurons (Roozendaal et al., 2009); previous work from our lab indicates that an upregulation of GluA1 in principal cells of the BLA may be responsible (Perusini et al., 2015).

Though the amygdala is likely to be the core structure in the stress circuit leading to PTSD, the hippocampus has also been shown to play an important role. MRI studies of PTSD patients frequently report substantial loss of gray matter in the hippocampus, though there is debate as to whether this is caused by the trauma or due to an underlying predisposition for diagnosis; in rodent studies, both circumstances have been observed (Bennett et al., 2015). Furthermore, in both human and rodent studies, the hippocampus is critical for the formation of a contextual memory (Fanselow, 2010), and for downstream association of that memory with emotional valence, as occurs during both normal contextual fear learning and after trauma (Orsini et al., 2011; Bennett et al., 2015). The hippocampus is also a major regulator of the HPA axis, where glucocorticoid receptor activation serves as a negative feedback mechanism of the acute stress response (Zhu et al., 2014; Herman et al., 2016).

Proper hippocampal functioning, particularly during contextual memory acquisition, depends upon cholinergic inputs originating from the medial septum in the basal forebrain cholinergic system. These inputs are critical for the formation of normal contextual associations, as blockade of muscarinic receptors by scopolamine in the hippocampus prevents contextual fear learning while leaving tone fear learning intact (Gale et al., 2001), and enhanced cholinergic tone during context exploration primes future contextual associations (Hersman et al., 2017). One known mechanism for muscarinic receptor effects on learning is through modulation of LTP, as receptor activation is known to facilitate hippocampal LTP (Burgard and Sarvey, 1990) and to be required for learning-related upregulation of AMPARs (Mitsushima et al., 2013). Acute stress leads to elevated hippocampal ACh (Stillman et al., 1997), and may induce hyperexcitation of cholinergic circuits (Zimmerman and Soreq, 2006) particularly in the hippocampus (Pavlovsky et al., 2012). Furthermore, acetylcholinesterase inhibitors in some cases induce psychopathologies very similar to PTSD (Kaufer et al., 1998). These converging lines of evidence point to cholinergic signaling as a critical aspect of plasticity during learning under stress, and perhaps as an important target for disruption of this enhanced plasticity in order to reduce the deleterious effects of stress and impair traumatic memory acquisition.

Animal models of aspects of PTSD such as stress-enhanced fear learning (SEFL) have recapitulated many aspects of human PTSD symptomology, such as resistance to extinction therapy (Long and Fanselow, 2012), and sensitization to future mild stressors (Rau et al., 2005). It also recapitulates the upregulation of glucocorticoid receptor expression observed in humans with PTSD (Labonté et al., 2014; Poulos et al., 2014), as well as suggesting molecular and cellular targets for future studies on traumatic stress (Ponomarev et al., 2010). In this model exposure to 15 inescapable foot shocks in one environment not only leads to high levels of fear to that environment, but to heightened levels of fear after a single shock in a novel environment, compared to animals that did not receive the 15 shocks. This sensitization to future mild stressors was not disrupted by extinction of the original traumatic context, nor by blockade of NMDARs during learning by icv infusion of 2-Aminophosphonovaleric acid (APV) before the trauma (Rau et al., 2005), suggesting circuit changes not strictly related to formation of an associative memory. This is also supported by the lack of requirement for memory of the trauma in juveniles for expression of the phenotype as adults (Poulos et al., 2014).

The relationship between cholinergic signaling during the trauma and manifestation of the SEFL phenotype is currently unknown. In our first experiment, we infuse scopolamine or aCSF vehicle into the dorsal hippocampus (DH) 1 h prior to SEFL, and demonstrate that scopolamine disrupts both trauma context memory formation and later sensitization of fear in a novel context. In our second experiment, we administer a tone-shock pairing instead of a context-shock pairing as the novel mild stressor, and demonstrate that scopolamine in DH does not block this sensitization, suggesting that cholinergic signaling in DH during trauma sensitizes future contextual learning, but not future learning about discrete stimuli. Finally, we provide evidence that these effects are not due to state-dependent effects of scopolamine administration, nor by blockade of consolidation, but are rather a consequence of blocking cholinergic-dependent contextual sensitization during a traumatic experience.

## Materials and Methods

### Subjects

A total of 64 naïve male Long-Evans rats, weighing 270–300 g (Harlan, Indianapolis, IN, United States) were individually housed and maintained on a 12-hour light/dark cycle with access to food and water *ad libitum*. Animals were handled daily (one-two min per rat) for at least 1 week prior to the start of all experimental procedures. The procedures used in this study were in accordance with policy set and approved by the Institutional Animal Care and Use Committee of the University of California, Los Angeles.

### Surgery

**FIGURE 1:**
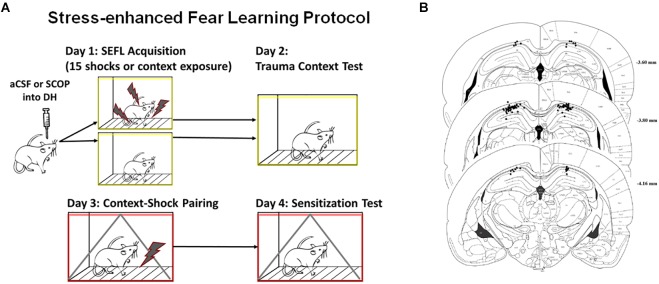
Stress-enhanced Fear Learning Design and Cannula Placements. **(A)** SEFL Context Procedure. Schematic representation of the stress-enhanced fear learning procedure, which in the SEFL group produces both high levels of fear in the trauma context as well as sensitization to a new context previously paired with shock. **(B)** Dorsal Hippocampus Cannula Placement. All hippocampal cannula tip placements are shown above. Rats with one or both internal tips outside DH were excluded from analysis (*n* = 65 included).

One week after housing, rats received surgical implantation of two guide cannulae aimed at the dorsal hippocampus. DH cannulae placements are shown in Figure 1B; rats with one or both cannulae tracts that missed the DH were not included in the behavior analysis. Rats were first anesthetized with sodium isoflurane (1–5%) and mounted in a Stereotaxic frame. Guide cannulae (26-gauge, 7 mm; Plastics One) were then lowered to the dorsal hippocampus (3.8 mm posterior to bregma, 2.5 mm lateral to bregma, and 1.8 mm ventral to dura). For infusions, the internal cannulae would extend 1.0 mm beyond the tip of the guide cannulae. Dental acrylic was used to fix cannulae to the skull, and dummy cannulae (33-gauge, 7 mm) were inserted into the guide cannulae.

### Apparatus

Behavioral training used a set of four identical fear conditioning chambers (30 × 25 × 25 cm, Med-Associates, Inc., St. Albans, VT, United States) equipped with a Med-Associates Video Freeze system. Individual boxes were enclosed in sound-attenuating chambers in an individual, dedicated experimental room. The SEFL context was comprised of chambers with aluminum sidewalls and a clear Plexiglas rear wall. The grid floor consisted of 16 stainless steel rods (4.8 mm thick) spaced 1.6 cm apart (center to center). Pans underlying each box were sprayed with a thin film of 50% Windex^®^ to provide the context with a scent. Chambers were individually lit from above by white lights and cleaned with 50% Windex in between squads. Fans mounted above each chamber provided background noise (60 dB). The experimental room was brightly lit with overhead white light. Animals were transported to the context in squads of eight in their home cages, which were slid onto hanging racks mounted to a portable cart and covered with a white sheet or black sheet. All aspects of the context were altered to create a distinctive single shock context. This context was comprised of an alternating large small or height-staggered grid floor and a black plexiglass A-frame. The context light was off, the experimental room light was red, and chambers were cleaned and scented with a 7% acetic acid solution. Rats were transported to the context in groups of 4 in a black tub with individual dividers and bedding on the floor. Many aspects of the context were altered again for the cohort of rats that received tone conditioning and tone test in a separate, third context. This context consisted of a white plexiglass floor, white curving plexiglass rear wall in order to make the chamber shaped like a semicircle, context light off, red and white experimental chamber lights concurrently on, and cleaned with Pine Sol. Groups of four rats were transported to this context together in a large transparent plastic tub with blue pads on the floor. All chambers were cleaned with a 10% bleach solution following each day of behavioral testing.

### Procedure

SEFL Context procedure is detailed in Figure 1A, while SEFL Tone procedure is detailed in Figure 3A. On Day 1, prior to the fear conditioning procedure, rats received bilateral infusions of either scopolamine hydrobromide (50 mg/ml concentration, 1 μl total volume in aCSF) or the same volume of aCSF, at a rate of 0.25 μl/min. This dose was chosen as it has been previously demonstrated to impair contextual fear acquisition without affecting tone fear acquisition or shock sensitivity (Gale et al., 2001). Rats were held by experimenters while injection cannulae (33-gauge; 8 or 10 mm), connected to 10-ml Hamilton syringes with PE-20 polyethylene tubing (Plastics One) and mounted on a microinfusion pump (Harvard Apparatus, South Natick, MA, United States) were inserted into the guide cannulae. Injection cannulae were left in place for an additional minute to facilitate diffusion. Dummy cannulae were then reinserted. Rats were returned to home cage for 1 h prior to being placed in conditioning chambers. Rats receiving the SEFL protocol received 15 unpredictable foot shocks (1 s, 1.0 mA) pseudorandomly spaced across a 90 min conditioning session. The first shock occurred after 3 min in the chamber. Rats receiving the No Stress (NS) protocol were placed in the context for 90 min but received no foot shocks. All rats were returned to their home cages after each experimental session. To assess the level of fear to the trauma context, on Day 2 rats were returned to the same context for a 5 min Trauma Context test. To assess the response to a novel stressor, on Day 3 rats were placed in a novel context. Three min after entering the context, they received a single foot shock (1 sec, 1.0 mA), and were removed from the context 1 min later. On Day 4, rats were returned to the single shock context and freezing was assessed over the 8 min Context Sensitization test. A subset of rats received the tone training protocol, which differed on Days 3 and 4. On Day 3, rats received a single tone presentation (80 dB, 20 s) co-terminating with a foot shock (1 mA, 1 s) after 3 min in the context. They were removed from the context 1 min after the shock. On Day 4, these rats were exposed to a novel third context, and received one tone presentation for a Tone Sensitization test. The use of a novel conditioning chamber to assess tone freezing reduces the contribution of contextual freezing to this measure. For the experiment to test whether scopolamine blocks consolidation of the trauma memory, we used a subset of DH rats who had previously received one shock in order to conserve research subjects. These rats then went on to experience the SEFL procedure, and received either aCSF or scopolamine directly following the trauma. Though this mild fear-inducing experience may subtly affect future fear learning, the use of only subjects who had received this experience minimized the between-group effects on variance. For assessment of fear, statistical analyses on freezing behavior were performed using an automated near infrared (NIR) video tracking equipment and computer software (Video Freeze, Med-Associates Inc., St. Albans, VT, United States).

### Histology

To assess cannulae placements, rats were anesthetized with isoflurane and decapitated. Brains were removed from the skull and placed in 10% formalin / 30% sucrose solution for 3 days prior to sectioning using a cryostat. Coronal sections (40 μm thick) were taken throughout the extent of the cannulae track and mounted on slides. Injection sites were reconstructed using bright field microscopy. Rats that had one or both cannulae or injector tracks outside the target structure were excluded from analysis (Figure 1B).

## Results

### Scopolamine in the DH Blocks Fear Acquisition to the Trauma Context

**FIGURE 2:**
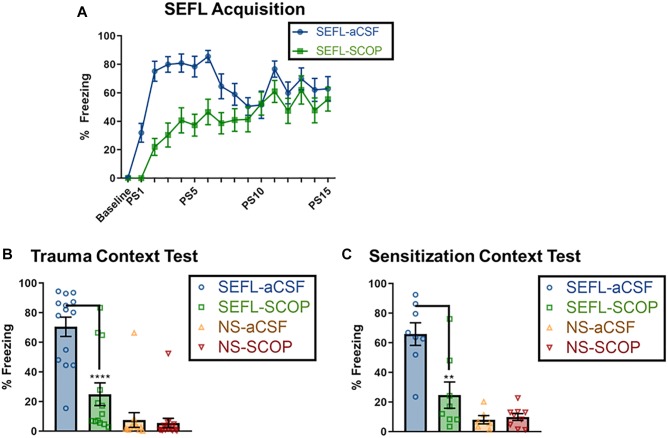
Scopolamine disrupts trauma memory formation and future sensitization. **(A)** SEFL Acquisition, where baseline represents the average freezing for the 3 min baseline, and “PS” designates post-shock freezing, as measured for 30 s beginning 30 s after the end of a shock. Non-shocked (NS) rats received a context exposure of the same length, but without any shocks; none exhibited freezing (data not shown). Both scopolamine and aCSF groups demonstrate post-shock freezing, though scopolamine rats have a slower rate of learning, as measured by lower levels of freezing toward the beginning of the session, but no difference by the end of the session (*n* = 15 per group). Data for the following two graphs are averaged across an 8 min test. **(B)** Stress Context Test. While SEFL-aCSF rats have very high levels of freezing, scopolamine attenuated the contextual freezing during the trauma test (*n* = 13 or more per group). **(C)** Sensitization Test. Rats that received one shock but no prior trauma (NS) show low freezing to the sensitization context. SEFL-aCSF rats show profoundly elevated freezing to the single shock; this freezing is attenuated by scopolamine administration prior to SEFL (SEFL-SCOP) (*n* = 6 or more per group). All individual data shown, error bars indicate SEM.

Naïve rats received an infusion of either scopolamine (SCOP) or vehicle (aCSF) into the dorsal hippocampus, and 1 h later experienced the 15-shock SEFL procedure or an equivalent context exposure with no stress (Figure 1A). As rats receiving no stress showed no freezing during the session and often fell asleep, their within-session freezing behavior was not analyzed. Of rats who received the SEFL procedure, scopolamine retarded fear acquisition, with the largest difference in freezing between drug and vehicle cohorts at the beginning of the session (Repeated Measures 2-Way ANOVA, Main effect of Drug [*F*(1,28) = 22.53, *p* < 0.0001], Main effect of Shock Number [Greenhouse-Geisser corrected, *F*(8.7,243.7) = 14.01, *p* < 0.0001], significant Interaction [Greenhouse-Geisser corrected, *F*(8.7,243.7) = 3.33, *p* < 0.0001], Sidak’s multiple comparisons test, difference between vehicle and SCOP groups from PS1 (*p* < 0.01) through PS6 (*p* < 0.05), but no difference on subsequent trials (Figure 2A). These results demonstrate that while scopolamine slows acquisition, it does not fully prevent freezing or within-session responding to a dangerous environment.

Though the effect of scopolamine infusion during trauma was mild, the effects on trauma memory recall were profound (Figure 2B). While vehicle rats demonstrated high fear to the trauma context (∼85% freezing), scopolamine led to a significant reduction in freezing in SEFL rats, but not in non-stressed controls [SEFL main effect (*F*(1,52) = 52.80, *p* < 0.0001), Drug main effect (*F*(1,52) = 17.68, *p* < 0.001), significant interaction between SEFL and Drug (*F*(1,52) = 14.71, *p* < 0.001); SCOP reduced fear in SEFL rats (*p* < 0.001) but not NS rats (*p* > 0.05)]. These findings suggest that cholinergic signaling in the DH, specifically at muscarinic receptors, is essential for the formation of a strong traumatic contextual memory.

### Scopolamine in DH Blocks Sensitization to a Novel Context CS

While scopolamine is sufficient to disrupt the memory of a traumatic experience, it was unknown whether this temporary disruption would affect future fear learning to a novel contextual stimulus. Off drug, these rats were exposed to a novel context and given a single shock, and contextual freezing was measured the following day in the same context to test contextual sensitization.

In vehicle rats, the prior stress enhanced subsequent fear learning to the new conditioning context, demonstrating the sensitizing nature of trauma to future fear learning (Figure 2C). However, scopolamine infusion prior to the traumatic event prevented this later sensitization [main effect of SEFL (*F*(1,27) = 32.37, *p* < 0.0001) and Drug (*F*(1,27) = 9.53, *p* < 0.01] and a significant interaction between SEFL and Drug [*F*(1,27) = 11.39, *p* < 0.01]; SEFL rats who received scopolamine froze significantly less than those that received vehicle (*p* < 0.01).

This blockade of future contextual sensitization is not an obvious consequence of blocking traumatic memory formation, as many manipulations that disrupt this trauma memory have no effect on later contextual sensitization (Rau et al., 2005; Poulos et al., 2014). Rather, this suggests a novel role of cholinergic signaling during the trauma for sensitization of contextual learning circuitry in the DH. Therefore, we next tested the contribution of cholinergic signaling in DH during trauma to sensitization to novel discrete cues, as the hippocampus plays little role on direct conditioning to discrete auditory cues (e.g., Kim and Fanselow, 1992; Anagnostaras et al., 1999).

### Scopolamine in DH Leaves Sensitization to Discrete Stimuli Intact

**FIGURE 3:**
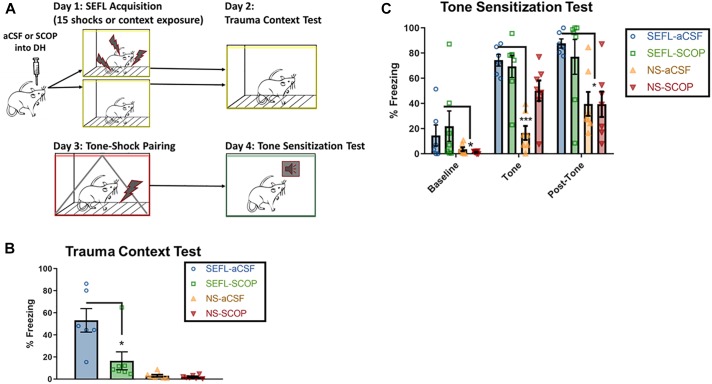
SEFL Tone Procedure and Tone Sensitization Test. **(A)** SEFL Tone Procedure. Schematic representation of the stress-enhanced fear learning procedure, which in the SEFL group produces both high levels of fear in the trauma context as well as sensitization to a new tone paired once with shock. **(B)** Trauma test after SEFL. In this cohort, we replicated our initial finding in the Trauma Test, namely that scopolamine attenuated freezing to the SEFL context. **(C)** Tone Sensitization after SEFL. After a tone shock pairing, there was some generalization to the tone-test context in both SEFL groups, but fear levels were low before tone onset. During the tone and in the post-tone period, all SEFL rats show elevated fear compared to rats that did not receive SEFL. These data indicate that while scopolamine into DH protects against future sensitization to contexts, it does not protect against future sensitization to tones (*n* = 6 or more per group). All individual data shown, error bars indicate SEM.

Instead of a context-shock pairing, a cohort of rats received a single tone-shock pairing after the SEFL procedure. The following day, they were exposed to a novel context and the tone was played, in order to test sensitization to discrete stimuli (Figure 3A).

Despite the blockade of fear to the trauma context, scopolamine during trauma did not prevent future sensitization to discrete cues (Figure 3B,C). Low baseline freezing was observed in both SEFL groups, due to low levels of contextual generalization to the novel, tone test context. During the tone, both groups of SEFL rats had a disproportionate freezing response and did not differ in their freezing levels (*p* > 0.05). Though some rats in the non-stressed scopolamine group had high initial freezing to the tone, which drove an interaction, the majority of non-stressed rats had very low responding to the tone [main effect of SEFL, *F*(1,23) = 28.63, *p* < 0.0001, no main effect of DRUG (*p* > 0.05), interaction, *F*(1,23) = 7.082, *p* < 0.05]. The disproportionate response of the SEFL groups continued in the period of time after the tone ended, while both non-stressed groups expressed normal levels of freezing [main effect of SEFL (*F*(1,23) = 17.1), *p* < 0.001), no main effect of DRUG (*p* > 0.05), no interaction (*p* > 0.05)]. Though non-stressed vehicle rats did significantly increase freezing levels between the tone and post-tone periods (*p* < 0.05), freezing remained below both SEFL groups at that time point (*p* < 0.05). Together, these data suggest that scopolamine in DH does not block all consequences of the traumatic experience. Rather, it has a selective effect on future contextual sensitization.

### Scopolamine Does Not Exert Effects Through State-Dependent Effects or Blockade of Consolidation

**FIGURE 4:**
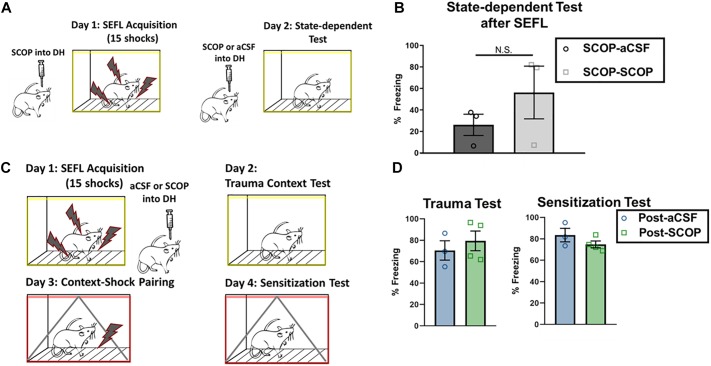
Test of state-dependency and effects on consolidation in DH. **(A,B)** DH cannulated rats that had received SEFL were given an infusion of scopolamine and re-tested in the trauma context (*n* = 3 per group). Rats that had previously received scopolamine did not freeze significantly more than rats that had previously received aCSF (unpaired *t* test, *p* > 0.05), suggesting that scopolamine’s disruptive effect on trauma test freezing after SEFL is not likely to be due to state-dependent effects. **(C,D)** DH cannulated rats that had previously experienced one shock were administered the SEFL protocol, and received post-SEFL administration of scopolamine or aCSF (*n* = 3 or more per group). Both groups showed similar levels of fear to the stress context 1 day later (*p* > 0.05) and the sensitization context 2 days later (*p* > 0.05). All individual data shown, error bars indicate SEM.

One potential mediator of the scopolamine disruption of freezing in the trauma context could be due to state-dependent effects, namely the incorporation of the mental attributes of experiencing scopolamine into the memory for the context. In order to test this, we subjected a subset of rats who had received scopolamine during SEFL to a second trauma test; this test occurred after an infusion of scopolamine (Figure 4A). No significant differences were seen between those that re-experienced the context under scopolamine or a vehicle infusion (Figure 4A,B; *p* > 0.05), suggesting that state-dependent effects are not the main mediator of the low levels of context fear seen in the scopolamine rats after SEFL.

Scopolamine could exert its effects on both trauma memory formation and contextual sensitization by disrupting memory formation, by disrupting consolidation, or by a combination of the two. Indeed, scopolamine is known to disrupt both acquisition and consolidation of contextual memory (Wallenstein and Vago, 2001). We tested this distinction by administering the SEFL protocol and infusing scopolamine or vehicle into the DH within 10 min after the protocol concluded (Figure 4C). Though prior intra-DH scopolamine infusion is disruptive for both the trauma test as well as for contextual sensitization after a single shock, post-SEFL scopolamine did not disrupt freezing in either test condition (Figure 4B,D; *p* > 0.05). Firstly, this test demonstrates that the reduced freezing levels seen during the sensitization test with pre-training scopolamine administration are not due to a prolonged duration of action of scopolamine, as post-training scopolamine did not disrupt freezing at that later time point. Secondly, this test is a conclusive demonstration that scopolamine in DH disrupts processes occurring during acquisition, but it does not exert its effects on trauma memory or later sensitization by blockade of memory consolidation.

## Discussion

The Stress-Enhanced Fear Learning (SEFL) procedure, in which an unpredictable 15-shock stressor is administered, generates a persistent sensitized state whereby novel mild stressors result in highly elevated levels of fear. This work extends the use of this model to study the role for cholinergic signaling during trauma on later sensitized responding, and demonstrates an important role for cholinergic signaling in DH for future contextual sensitization.

Pre-training administration of scopolamine into DH prevented contextual fear memory formation for an intense, 15-shock stressor. Though cholinergic signaling in DH has long been known to facilitate contextual processing, this is the first demonstration that even intense trauma cannot overcome the blockade of memory formation by scopolamine.

In addition to its role in contextual memory formation, this work has demonstrated a novel role for cholinergic signaling at muscarinic receptors in DH during a traumatic event for later contextual sensitization. Scopolamine administration into DH prior to the traumatic event blocked future contextual sensitization to a mild, single context-shock pairing in the absence of drug. The blockade of trauma memory formation is unlikely to be the mechanism for blocking this contextual sensitization for a number of reasons. The first is that blockade of trauma memory formation by blocking NMDA receptors, extinction of the trauma memory (Rau et al., 2005), and forgetting of trauma memory (Poulos et al., 2014), do not eliminate contextual sensitization to novel mild stressors. The second is that blockade of traumatic memory formation by scopolamine does not eliminate future sensitization to discrete stimuli. The selectivity of this blockade to contextual sensitization, while leaving discrete stimulus sensitization intact, points to a direct effect on the processing of contextual cues.

The mechanism by which scopolamine in DH disrupts this cholinergic-dependent sensitization was not a focus of this study, but some noted effects of scopolamine can be ruled out. For example, though scopolamine has been shown to affect consolidation processes, these effects are not sufficient to block contextual sensitization, as post-SEFL scopolamine infusion had no effect on later sensitization. Another noted effect of scopolamine, though after systemic administration, was an upregulation of M1 cholinergic receptors, alpha 7-containing nicotinic receptors, and NMDAR1 glutamate receptors in the DH (Falsafi et al., 2012), however, these effects would also occur with post-SEFL scopolamine, so are not sufficient for blockade of contextual sensitization. Finally, one effect of systemic infusion of scopolamine in an increase in hippocampal ACh release; blockade of nicotinic receptors reduced both this release and working memory task impairment in another study (Newman and Gold, 2016). This is unlikely to be a primary mediator of the scopolamine blockade of contextual sensitization for a couple reasons. The first is that this is a working memory task, rather than contextual encoding task, which may have differential hippocampal requirements (von Engelhardt et al., 2008). The second is that increased hippocampal ACh release alone promotes, rather than impairs, contextual encoding (Hersman et al., 2017). This potential elevated ACh alone, therefore, is insufficient to explain the results.

Multiple mechanisms likely play a role in this cholinergic-dependent sensitization. During acute stress, ACh levels in DH become profoundly elevated (Mark et al., 1996) and co-vary with corticosterone levels (Mitsushima et al., 2008). This acute stress leads to long-lasting gene expression changes that are dependent upon muscarinic receptor activation (Kaufer et al., 1998). In the case of chronic stress, this leads to long-lasting hypersensitivity to ACh, as measured by increased CA1 pyramidal neuronal glutamate release in response to muscarinic receptor activation (Pavlovsky et al., 2012), though whether this applies to a single traumatic stressor is unknown. The fact that acetylcholinesterase inhibitors, which lead to elevated ACh levels, can in some cases lead to psychopathologies reminiscent of PTSD (Rosenstock et al., 1991; McLay and Ho, 2007) supports the notion that elevated cholinergic tone during an acute, traumatic stressor plays a role in the sensitized responding seen in human post-traumatic stress.

Though ACh has direct effects on hippocampal LTP, contextual sensitization may occur through other mechanisms, leading to a dissociation between the memory-impairing but sensitization-sparing effects of APV and the cholinergic effects on both processes. For example, muscarinic receptor activation on CA1 pyramidal cells altered spine morphology (Schätzle et al., 2011), while activation on CA1 parvalbumin cells led to enhanced action potential frequency and facilitated GABAergic transmission (Bell et al., 2015); both these effects were NMDA-independent, yet have the potential to alter future circuit dynamics and memory formation. Another mechanism for sensitization of hippocampal circuitry would be the known consequences of muscarinic receptor activation, which can lead to hippocampal glucocorticoid receptor downregulation, NMDA-receptor downregulation, and elevated corticosterone levels (Hoeller et al., 2016). In this study, these effects were not dependent upon NMDA-receptor activation, which is a compelling parallel to the inability of APV to block the SEFL phenotype. Further work is needed to understand the precise contribution of muscarinic receptor activation in the hippocampus to mechanisms of contextual sensitization.

Though scopolamine in DH blocks contextual sensitization, it does not block all consequences of the traumatic experience. With the known contribution of BLA GluR1 receptors and glucocorticoid signaling to the SEFL phenotype (Perusini et al., 2015), it may be the case that stress-induced changes in BLA lead to the continued persistence of tone sensitization after DH scopolamine. Rather, scopolamine in DH isolates and blocks a particular consequence of trauma, the sensitization of future contextual learning.

In addition to the direct modulation of hippocampal processing, cholinergic blockade in DH may disrupt inter-regional communication necessary for future contextual sensitization after trauma. Scopolamine administered systemically disrupts resting state functional connectivity between mouse brain regions involved in memory (Shah et al., 2015), and this disruption of inter-regional communication, particularly between the DH and BLA, may play a role in its profound effects. This interaction between cholinergic signaling and inter-regional communication is evident in humans, as individual differences in cholinergic gene expression mediate functional connectivity differences in humans between the basal forebrain, amygdala, and hippocampus during processing of emotional stimuli (Gorka et al., 2015). This interaction may become dysregulated after trauma due to hyperactive cholinergic circuits. Soldiers with PTSD (compared to those without) have inter-regional hypersynchrony at high frequencies (80–150 Hz), as well as a decrease in signal variability; most evident in the network containing hippocampus and amygdala (Mišiæ et al., 2016).

Anxiety disorders such as PTSD may present with symptoms related to discrete stimulus sensitization, contextual sensitization, or both, however, the evidence that these symptoms may be produced by different patterns of neural activity was previously lacking. This study demonstrated for the first time a selective contribution of cholinergic signaling during trauma exposure to future contextual, rather than general, sensitized responding. For individuals who primarily overreact to unpleasant contexts, this dissociation could provide a basis for the development of specialized treatments. Even in the case where sensitized responding to contexts and discrete stimuli are both observed, pharmacological targeting of contextual circuitry could help to normalize responding to discrete stimuli, by facilitating improved discrimination between safe and unsafe contexts (Chen and Etkin, 2013; Glenn et al., 2018).

This work highlights the important contribution of hippocampal signaling to the sensitization that occurs after an acute traumatic event and provides the first evidence that cholinergic signaling during the trauma may induce changes leading to enhanced contextual sensitization after trauma. Future work will endeavor to isolate the mechanism by which this signaling changes hippocampal processing and understand the contribution of cholinergic signaling during trauma to future anxiety states, hopefully leading to novel treatments for stress-induced sensitized states.

## Data Availability

All datasets generated for this study are included in the manuscript and/or the supplementary files.

## Author Contributions

SH designed the experiments, performed surgeries, organized and ran behavioral experiments, analyzed results and was the primary writer of the manuscript. AH ran experiments and assisted with analysis and development of figures. LH and SS performed some surgeries and ran behavioral experiments. JL and AP ran behavioral experiments. MF assisted with design of experiments, analysis of data, and writing of the manuscript.

## Conflict of Interest Statement

MF is on the scientific advisory board of Neurovation, Inc. The remaining authors declare that the research was conducted in the absence of any commercial or financial relationships that could be construed as a potential conflict of interest.
